# Fatty Liver Index Predicts Further Metabolic Deteriorations in Women with Previous Gestational Diabetes

**DOI:** 10.1371/journal.pone.0032710

**Published:** 2012-02-29

**Authors:** Latife Bozkurt, Christian S. Göbl, Andrea Tura, Marek Chmelik, Thomas Prikoszovich, Lana Kosi, Oswald Wagner, Michael Roden, Giovanni Pacini, Amalia Gastaldelli, Alexandra Kautzky-Willer

**Affiliations:** 1 Unit of Gender Medicine, Division of Endocrinology and Metabolism, Department of Internal Medicine III, Medical University of Vienna, Vienna, Austria; 2 Division of Feto-Maternal Medicine, Department of Gyneacology and Obstetrics, Medical University of Vienna, Vienna, Austria; 3 Metabolic Unit, Institute of Biomedical Engineering, National Research Council, Padova, Italy; 4 High Field Magnetic Resonance Centre of Excellence, Medical University of Vienna, Vienna, Austria; 5 Chemical Laboratory Diagnostics, Clinical Institute for Medical, Medical University of Vienna, Vienna, Austria; 6 Department of Metabolic Diseases, German Diabetes Center, Leibniz Center for Diabetes Research, Institute for Clinical Diabetology, Heinrich-Heine University, Düsseldorf, Germany; 7 Cardiometabolic Risk Unit, Institute of Clinical Physiology, National Research Council, Pisa, Italy; Virgen Macarena University Hospital, Spain

## Abstract

**Background and Aims:**

Determinants of fatty liver (FL) might be predictive for further deterioration in insulin resistance (IR) in women with previous gestational diabetes (pGDM). The aim was to evaluate the association between pGDM, FL and future manifestation of type 2 diabetes (T2DM) by a detailed pathophysiological characterization early after pregnancy.

**Methods:**

68 pGDM and 29 healthy controls were included 3–6 months after delivery and underwent specific metabolic assessments: status of IR was determined via oral- and intravenous-glucose-tolerance-tests with analysis of proinflammatory factors and kinetics of free-fatty-acids (FFA). According to the fatty-liver-index (FLI), pGDMs were categorized into three groups with low (FLI≤20), intermediate (20<FLI<60) and high (FLI≥60) risk for FL to assess differences in metabolic parameters at baseline as well as in the 10-year incidence for T2DM. Accuracy of FLI was proven with 1H-magnetic-resonance-spectroscopy.

**Results:**

FL was strongly associated with IR in pGDM. pGDM with FLI≥60 showed significantly increased interleukin-6, plasminogen-activator-inhibitor-1, tissue-plasminogen-activator, fibrinogen and increased ultrasensitive-C-reactive-protein compared to the low risk group (FLI≤20). Analysis of FFA indicated a less pronounced decrease of plasma FFA levels during the oral-glucose-tolerance-test in subjects with FLI≥60. History of GDM plus FLI≥60 conferred a high risk for the manifestation of diabetes over 10 years of observation as compared to pGDMs with FLI≤20 (HR:7.85, Cl:2.02–30.5, p = 0.003).

**Conclusion:**

FL is closely linked to GDM, especially to IR and inflammation. Most interestingly, subjects with the highest FLI values showed significant alterations in FFA kinetics and a higher risk to develop T2DM in future.

## Introduction

Most of the important risk determinants of the metabolic syndrome are present in women with previous gestational diabetes (pGDM) and indicate their high risk for developing type 2 diabetes and cardiovascular disease in later life [1]. Fatty change of the liver is more and more emerging as an early marker of mechanisms that predispose to future metabolic events. Nonalcoholic fatty liver disease (NAFLD) is therefore suggested to represent another feature of the metabolic syndrome with insulin resistance (IR) as the key linking factor [2].

Generally, it is suggested that the presence of NAFLD in insulin resistant subjects implies more severe systemic oxidative stress and endothelial dysfunction and thus predisposes to arteriosclerosis and cardiovascular disease [3], [4]. History of GDM is accepted as a representative model for studying very early metabolic alterations that might precede manifestation of type 2 diabetes and cardiovascular disease specifically in women. We and others have previously shown a strong correlation between IR and/or obesity and different adipokines or markers of fibrinolytic dysfunction, subclinical inflammation and atherogenesis in women with pGDM [5], [6]. In a small cohort compared to controls we demonstrated a slight decrease of muscle mitochondrial function in pGDM with no difference in ectopic fat accumulation in skeletal muscle but with hepatic fat five years after index pregnancy [7].

Studies linking NAFLD with incident type 2 diabetes are limited but indicate that NAFLD or raised liver enzymes could improve prediction of diabetes beyond other established prognostic factors [8]. However, diagnosis of NAFLD is regarded as clinically problematic due to the invasive character of the gold-standard method of liver biopsy [8]. Bedogni et al introduced the Fatty Liver Index (FLI), a multivariate model including biomarkers to accurately estimate presence of fatty liver. This estimation has already been applied by scientific investigations including large populations to determine the prevalence of fatty liver [9]–[13].

Thus, the primary objective of the present study was to evaluate the relationship between pGDM representing a special cohort of early prediabetes in females and the risk for presence of fatty liver, estimated by the FLI, with special regard to insulin resistence as well as the association with prothrombotic and proinflammatory factors as well as the free-fatty-acid (FFA) kinetics during an oral-glucose-tolerance-test (OGTT) at 3 to 6 month after index pregnancy. Additionally, we prospectively examined the association of FLI early after pregnancy with future development of type 2 diabetes within an observed period of 10 years.

## Methods

### Subjects

The study design was previously reported in detail [8]. Briefly, women with pGDM (n = 68) were prospectively recruited 3–6 months after delivery at our diabetes outpatient clinic (Department of Internal Medicine III, Medical University of Vienna). GDM was diagnosed according to the criteria of the 4^th^ International Workshop conference on GDM [14]. A total of 29 women comparable for age, but without any risk for diabetes and with normal glucose tolerance during pregnancy were included prospectively and served as a control group (NGT).

At enrollment, all women with prior GDM received general information about their risk of diabetes and potential prevention strategies, including dietary advice and instructions on physical exercise, as recommended.

Thereafter, pGDMs were annually invited for reexaminations (OGTT) for the following 10 years. A manifestation of overt diabetes was diagnosed, if fasting plasma glucose levels or 2 h-OGTT levels exceeded 126 mg/dl or 200 mg/dl in addition to clinical symptoms, respectively.

All subjects gave written informed consent for participation in the study. The study was approved by the local ethics committee (Ethics Committee of the Medical University of Vienna) and was performed in accordance with the Declaration of Helsinki.

### Metabolic assessments

At baseline, medical data (i.e., medical history, drug use) of all eligible women were collected according to a standardized questionnaire. Weight, height, and waist circumference were obtained at enrolment according to a standardized protocol.

Subjects underwent a frequently sampled intravenous-glucose-tolerance-test as well as a 2 h-75 g OGTT 3–6 months after delivery. All women ingested an isocaloric diet containing 200 g of carbohydrates/day and refrained from exercise for at least 3 days before the studies. Metabolic tests were performed on different days within the same week after 10- to 12-h overnight fasting.

For frequently sampled intravenous-glucose-tolerance-test, which was performed after the OGTT, glucose (300 mg/kg body weight) was infused for 30 seconds starting at time 0; then, normal insulin (0.03 U/kg, Humulin R; Eli Lilly, Indianapolis) was infused from 20 to 25 min. Venous blood samples for determination of plasma concentration of glucose, insulin and C-peptide were collected before glucose infusion (fasting sample) and at 3, 4, 5, 6, 8, 10, 14, 19, 22, 27, 30, 35, 40, 50, 70, 100, 140 and 180 min afterwards.

Minimal model analysis of plasma glucose and insulin concentrations provided the insulin sensitivity index (S_I_), which describes the insulin effect on glucose disappearance [15]. In order to accurately evaluate associations of FLI to insulin resistance, frequently sampled intravenous-glucose-tolerance-test data were used to divide pGDM into subgroups with (pGDM-IR, n = 25) and without (pGDM-IS, n = 37) impaired insulin sensitivity (Table 1) according to the cutoff value for insulin sensitivity index (obtained from the frequently sampled intravenous-glucose-tolerance-test): S_I_ = 2.8 10^−4^ min^−1^/(µU/ml) [16].

**Table 1:** Characteristics of pGDM subjects grouped by insulin resistance status (means±SD).

	n	NGT	n	PGDM-IS	N	PGDM-IR	p-value
Age (years)	29	30.5±5.2	37	32.8±4.2	25	32.5±5.7	0.133
Waist (cm)	29	80.8±15.9* †	37	89.0±12.3§	25	97.4±15.5	<0.001
BMI (kg/m^2^)	29	25.4±6.4†	37	25.4±4.1§	25	30.4±5.4	<0.001
TG (mg/dl)	29	89.7±44.8	37	106.4±68.4	25	119.5±51.1	0.101**
GGT (U/l)	29	11.3±10.7*	37	7.32±3.8§	25	10.6±5.01	0.014**
FLI	29	20.1±29.0†	37	19.2±19.9§	25	45.9±32.3	<0.001**
ALT (U/l)	29	15.8±15.2	37	16.6±14.9	25	17.3±11.5	0.495**
AST (U/l)	29	13.2±8.2	37	11.1±6.0	25	11.7±5.6	0.381**
Fasting glucose(mg/dl)	29	83.8±8.3†	37	88.4±12.7§	25	95.8±16.2	0.003
HbA1c (%)	29	5.17±0.2* †	36	5.42±0.4§	25	5.63±0.5	<0.001
OGIS (ml min^−1^ m^−2^)	29	486.5±71.7†	36	466.1±91.7§	23	406.4±80.9	0.003

Triglycerides (TG), gamma-glutamyltransferase (GGT), glycosylated haemoglobin-A1C HbA1c, body mass index BMI, oral glucose insulin sensitivity index OGIS, fatty liver index (FLI), alanine aminotransferase (ALT) and aspartate aminotransferase (AST).

p-values were determined by one-way analysis of variance.

*p<0.05 NGT vs. pGDM-IS;

† p<0.05 NGT vs. pGDM-IR;

§ p<0.05 pGDM-IS vs. pGDM-IR;

**p-values are based on log-transformed data.

Insulin sensitivity was additionally estimated from the OGTT by the oral glucose insulin sensitivity index (OGIS) [17].

### Fatty liver Index (FLI)

Presence of fatty liver was evaluated using the recently validated FLI [9]. The index was calculated based on data of visits at 3–6 months after delivery in pGDM (n = 68) and NGT (n = 29) subjects. Comparable to the generally used diagnostic method of ultrasound, the accuracy FLI in detecting fatty liver was given with 0.84 (95% confidence interval [CI] 0.81–0.87) [9]. The index uses an algorithm based on body-mass-index (BMI), waist circumference (WCF), triglycerides (TG), gamma-glutamyl transferase (GGT), and natural logarithm (ln) as follows: FLI = exp[0.953×ln(TG)+0.139×BMI+0.718×ln(GGT)+0.053*WCF−15.745]/(1+exp[0.953*ln(TG)+0.139×BMI+0.718×ln(GGT)+0.053*WCF−15.745])×100.ngs

Our group based analysis was performed according to Gastaldelli et al [10]. Women with pGDM were divided into three groups: G1 with FLI≤20, who have a very low risk for fatty liver; G2 with 20<FLI<60, an intermediate group; and the high risk group G3 with FLI≥60.

### Accuracy of FLI

The accuracy of FLI was tested in a subgroup of prospectively included young female subjects (17 with pGDM, 8 with NGT during pregnancy) who underwent venous blood sample collection and ^1^H-magnetic resonance spectroscopy (MRS) of the liver [7]. ^1^H-MRS measurements were performed by a 3-T whole body spectrometer (Bruker Biospin, Ettlingen, Germany). Hepatocellular lipid content was quantified from localized ^1^H spectra using the series stimulated echo acquisition mode (STEAM) sequence within a volume of interest of 27 cm^3^ [18].

### Laboratory methods

Insulin (Serono Diagnostics), C-peptide (CIS Bioe International) and total leptin (Linco) were quantified in duplicate by radioimmunoassay with interassay coefficients of variation of <5.5%. Total adiponectin was measured in duplicate in fasting plasma samples using an ELISA system (Osaka University, Japan). Plasma concentrations of active plasminogen-activator-inhibitor-1 (PAI-1) antigen were measured by Actibind PAI-1 ELISA (Technoclone) and tissue-plasminogen-activator (t-PA) antigen concentrations by Coaliza t-PA-ELISA Kit (Chromogenix AB). Tumor-necrosis-factor (TNF)-α was measured by a quantitative sandwich enzyme immunoassay technique (Quantikine HS Immunoassay kit), interleukin-6 by ELISA systems (R&D Systems), and ultrasensitive C-reactive protein (usCRP) by means of particle enhanced immunonephelometry (N High Sensitivity CRP Reagent, BN Systems). FFA were measured by microfluorimetry (Wako Chem USA Inc.).

### Statistical analysis

Data are given as means±SD and 95% confidence intervals (CI). In case of skewed distributed variables (usCRP, TNF-α, interleukin −6, TG, GGT, AST, ALT and FLI), a log transformation was applied. Comparisons among NGT, IS-pGDM and IR-pGDM subjects as well as between FLI grouped pGDMs were performed with Fisher protected least significant difference tests. These post-hoc tests are only calculated in the case of significant global tests, what is accepted as the appropriate procedure to deal with multiplicity in the case of three groups. Because of the lower sample size, comparisons of adiponectin were based on Kruskal-Wallis-test and the exact version of the Wilcoxon test. Differences of categorical variables were analyzed by the Chi-square test.

The association between FLI and hepatocellular lipid content, measured by MRS, was analyzed using Pearson's product-moment correlation (r_P_) and approved by nonparametric Spearman's rank correlation as well as polynomial regression models.

Linear mixed effects models including an interaction term for FLI groups, were used to assess differences in FFA changes during the 2 h-OGTT depending on the extent of fatty liver disease. The identification number of the patients was included as a random effect.

Odds ratios were computed by logistic regression to estimate the predictive value of FLI on early onset of prediabetes (impaired fasting glucose and impaired glucose tolerance) or diabetes at baseline (within one month after recruitment). In addition, Cox proportional hazard models were used to analyze if different FLI groups were predictive for the manifestation of diabetes over 10 years of follow-up.

Statistical analysis was performed using R (V2.11.1). A two-sided p-value≤0.05 was considered statistically significant. No adjustment for multiple statistical testing was performed.

## Results

### Association between FLI and hepatocellular lipid content

**Figure 1:**
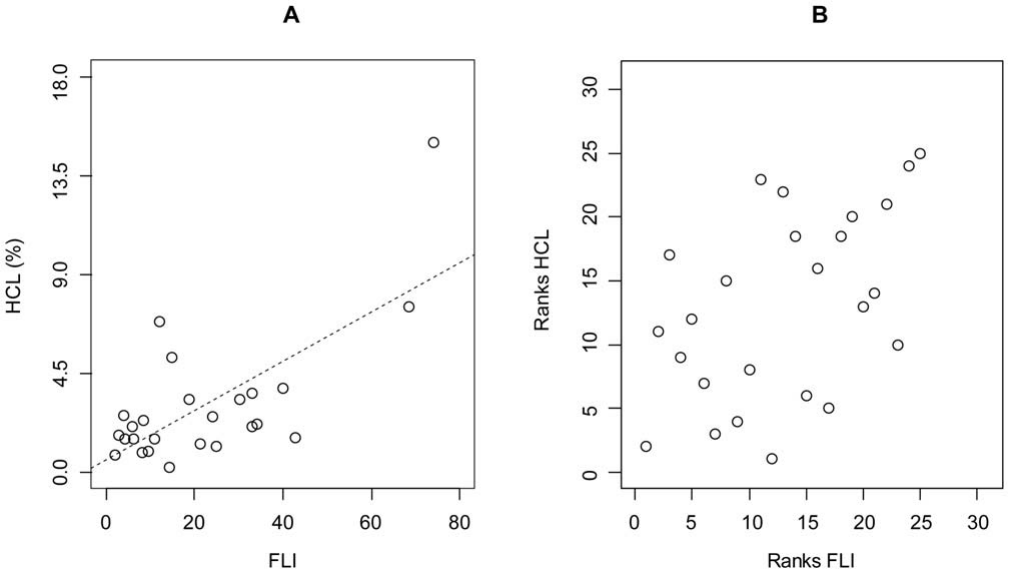
Association between hepatocellular lipid content and FLI based on regular parametric (A) as well as a ranks based nonparametric correlation (B).

We found a strong correlation between FLI and hepatocellular lipid content (r_p_ = 0.70, p<0.001, R^2^ = 0.47), approved by nonparametric statistical analysis (r_S_ = 0.50, p = 0.011). There appeared no interaction between the NGT and the pGDM subgroup. In comparison to the linear estimation, R^2^ showed a strong increase after modeling a quadratic association between FLI and hepatocellular lipid content (R^2^ = 0.65) and the equation was even further improved (R^2^ = 0.76) by including a cubic function into the model, which explained 76% of the variance. The scatter plot describing the association between hepatocellular lipid content and FLI is given in Figure 1. Therefore, we suggest a robust nonlinear relationship between FLI and hepatocellular lipid content measured by ^1^H-MRS, what is in accordance with the cut-points of Gastaldelli *et al.* [10].

### Association of fatty liver with IR and proinflammatory factors

**Figure 2:**
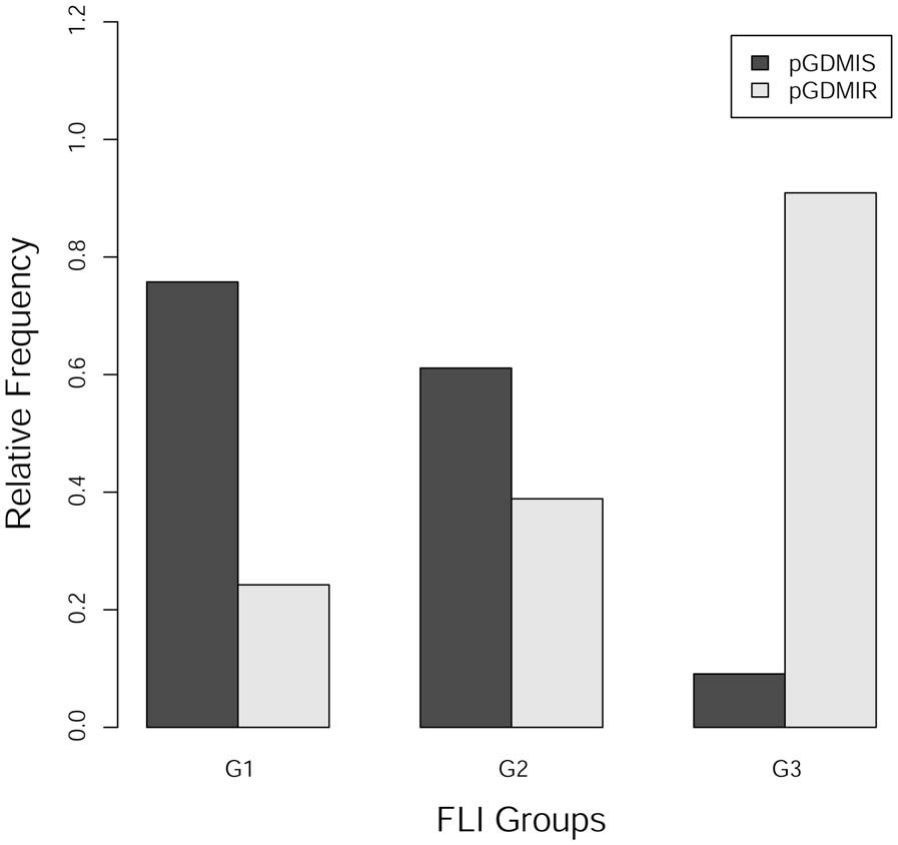
Association of FLI groups with status of insulin resistance. Degree of insulin resistance as defined by frequently sampled intravenous-glucose-tolerance-test (IR: SI<2.8 10−4 min−1/(µU/ml)) in FLI risk groups G1 (low risk), G2 (intermediate risk) and G3 (high risk) given as relative frequencies.

Descriptive characteristics as well as comparisons of parameters included in the FLI between NGT, IS-pGDM and IR-pGDM subjects are given in Table 1. Whereas ln(FLI) values of NGT and IS-pGDM women were comparable (p = 0.104), IR-pGDM subjects showed higher levels as compared to both NGT (p<0.001) and IS-pGDM (p = 0.006) women. In particular, risk for fatty liver as categorized by FLI cut-points was closely related to the status of IR (p<0.001) estimated by frequently sampled intravenous-glucose-tolerance-test (Figure 2). Particularly, group G3 showed significantly lower oral glucose insulin sensitivity index (OGIS) levels as compared to G1 or G2, an effect remaining also significant after adjustement for BMI and waist circumference by analysis of covariance. In a sensitivity analysis the time difference between delivery and testing was neither associated with FLI nor with parameters of insulin sensitivity.

Table 2 shows comparisons of FLI grouped pGDM women. We observed lower adiponectin (p = 0.039) and higher leptin levels (p<0.001) in pGDM of group G3, as compared to G1. When compared to G1, G3 women exhibited increased values of inflammation markers usCRP (p = 0.005) and interleukin-6 (p = 0.006), in addition to higher PAI-1 (p<0.001), t-PA (p<0.001) and fibrinogen (p<0.001). Total numbers of parameters are presented in table 2, and are partly restricted due to random missing of some analyses. However, with exception of adiponectin the drop out rates are at moderate level and violation of statistical comparisons could be excluded.

**Table 2:** Comparison of metabolic parameters within fatty liver index (FLI) groups in pGDM (means±SD).

	n	G1	n	G2	n	G3	p-value
Age (years)	33	32.0±4.4	20	32.6±4.3	15	34.5±6.1	0.242
Waist (cm)	33	82.6±7.1* †	20	97.0±8.0§	15	112.2±12.2	<0.001
BMI (kg/m^2^)	33	24.0±3.0* †	20	28.9±2.2§	15	35.5±4.8	<0.001
Fasting glucose (mg/dl)	33	85.9±10.7* †	20	95.0±15.0§	15	105.6±17.8	<0.001
Adiponectin (µg/ml)	23	6.95±2.56†	13	5.37±2.27	5	5.34±2.44	0.048
Leptin (ng/ml)	31	10.6±5.8* †	18	16.7±6.2§	12	25.6±10.2	<0.001
TNF-α (pg/ml)	26	5.22±6.9	15	6.91±7.6	12	8.09±9.4	0.286**
IL6 (pg/ml)	27	2.07±2.3†	13	14.5±43.8	13	5.40±3.3	0.021**
PAI-1 (ng/ml)	32	2.80±0.7* †	20	3.30±0.4	13	3.73±0.8	<0.001
t-PA (ng/ml)	32	3.30±1.8†	20	4.34±1.3	13	5.57±2.6	0.002
Fibrinogen (mg/dl)	32	330.7±54.6†	20	346.9±55.7§	13	422.0±72.2	<0.001
OGIS (ml min^−1^ m^−2^)	32	480.7±84.2* †	17	434.3±74.6§	13	344.5±44.1	<0.001
FFA at fasting (µmol/ml)	30	540.8±212.8	10	617.5±120.6	13	607.4±182.6	0.414
FFA at 60 min (µmol/ml)	30	94.1±42.2†	10	142.5±68.2§	13	304.4±176.1	<0.001
usCRP (mg/dl)	27	0.43±0.8†	16	0.42±0.6	13	0.71±0.5	0.019**

G1 = FLI≤20, G2 = 20<FLI<60 and G3 = FLI≥60;

Tumor-necrosis-factor (TNF-α), interleukin-6 (IL-6), plasminogen-activator-inhibitor (PAI), tissue-plasminogen-activator (t-PA), oral-glucose-insulin-sensitivity-index (OGIS), free-fatty-acids (FFA), and ultra-sensitive c-reactive protein (usCRP). Data are mean±SD. Data on adiponectin are referred as median and interquartil ranges.

p-values were determined by one-way analysis of variance or Kruskal-Wallis Test, respectively.

*p≤0.05 G1 vs. G2;

† p≤0.05 G1 vs. G3;

§ p≤0.05 G2 vs. G3;

**p-values are based on log-transformed data.

### Association of fatty liver and FFA kinetics

**Figure 3:**
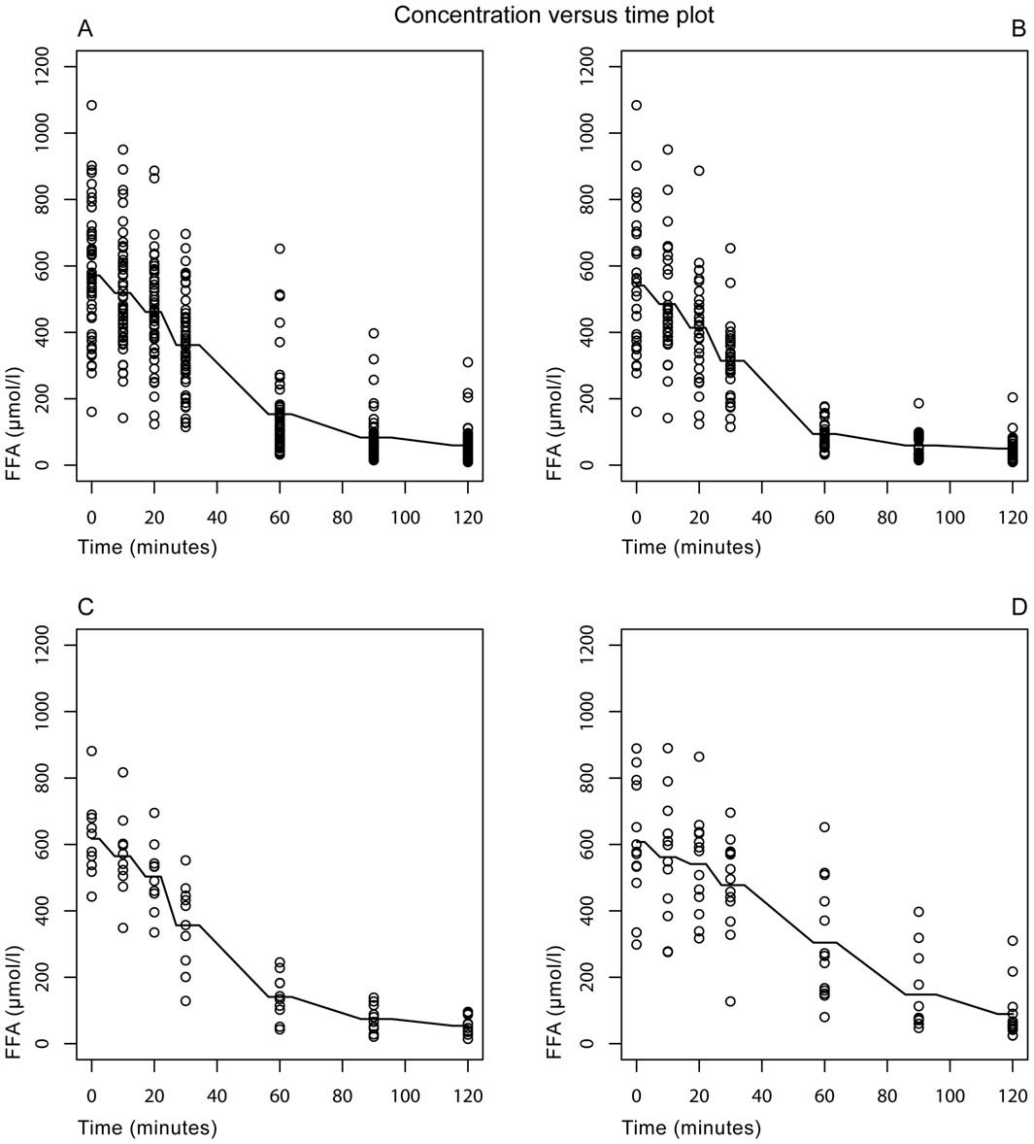
*Kinetics of free-fatty-acids (FFA).* Concentrations of free fatty acids (FFA) during the 2 h-75 g-oGTT in the total sample (A) as well as in the FLI groups: FLI≤20 (B), FLI 20–60 (C), FLI≥60 (D).

As given in Figure 3, FFA levels were similarly suppressed at 60 min in both women of group G1 and G2, in contrast to G3. Indeed, we found a significant FLI-group-by-time interaction using linear mixed effects models, suggesting a less pronounced decrease in FFA levels in G3 women as compared to G1 (B = 2.53, CI:1.15–3.92, p<0.001). Thus, FFA levels at 60 min were higher in G3 women, despite comparable baseline levels in cross-sectional analyses.

### Association of fatty liver with diabetes incidence

Logistic regression revealed that the incidence of prediabetes or diabetes within the first month after recruitment increased with FLI levels (OR:1.05, CI:1.02–1.08, p<0.001). The median time to diagnosis was 2.5 years (IQR: 1–4 years). In Cox proportional hazard models FLI levels were significantly associated with a higher risk of diabetes manifestation over 10 years of observation (HR:1.03, CI:1.01–1.05, p = 0.002). Subjects in G3 (7 of 13 women (53.8%) showed an even more pronounced risk for overt diabetes as compared to women defined as G1 (3 of 32 women, 9.4%, HR:7.85, CI:2.02–30.5, p = 0.003). Out of 16 NGT subjects with OGTTs available during follow-up, no case of overt diabetes or impaired glucose tolerance was detected. In pGDM participants, a second pregnancy after the index pregnancy was reported only in 12 women. Ten of them again developed GDM (83%), but there were no associations with FLI groups in descriptive analysis. The median changes in body weight were not significantly different between subjects who developed overt diabetes during follow-up (median: 1.5 kg, IQR: 8.2) and those who did not (median: 1.0 kg, IQR: 7.5) but also not between subjects of different FLI groups (G1: 0.0 kg, G2: 1.3 kg, G3: 1.0 kg).

## Discussion

Our results suggest that insulin resistant women with recent GDM, have an increased risk for presence of fatty liver. This risk might be of relevance, as fat accumulation in the liver not only represents the starting point for liver injury in IR but may also be a condition with far-reaching metabolic consequences: from diabetes to increased cardiovascular morbidity [4]. Moreover, we found that fatty liver is associated with parameters of subclinical inflammation in women with pGDM and demonstrate the predictive value of the FLI for diabetes manifestation. The new concept for the genesis of metabolic disorders in obesity, where fatty liver was suggested to be a better predictor of IR and atherosclerosis than visceral fat, is based on this theory [19], [20].

A sex-specific impact of fatty liver is assumed as women with NAFLD are more impaired in IR than men compared to their healthy counterparts [21], [22]. In a follow-up study of the KORA population, including men and women from the general German population with a wide age range, logistic regression models including age showed that male participants with fatty liver as defined by the FLI had an OR of 3.21 for diabetes whereby women had an increased OR of 7.92 compared to the healthy reference group [12]. However, the underlying mechanisms are still unclear. With our study, we have shown that the level of FLI relates to degree of IR, as IR-pGDM had significantly higher FLI values than women with NGT or IS-pGDMs. This is observed for the first time in a group of pGDM women, who were examined within the same time period early after gestation and were followed-up for 10 years. Our data are corroborated by a recent cross-sectional study where women affected by GDM within the last 10 years were demonstrated to have a higher prevalence of NAFLD compared to women with no history of GDM but after excluding those pGDM who developed overt diabetes [23]. Coherently, another group of women with increased insulin resistance, namely those with polycystic ovary syndrome, were recently shown to have higher FLI values than control women [13].

The pathogenesis of NAFLD is unknown, and it was suggested that adipose tissue IR could promote hepatic steatosis by increased FFA flux to the liver [24] and the consequent increase in inflammation and oxidative stress (multiple-hits hypothesis [25]), whereby both may determine liver injury by altering hepatic mitochondrial function [26]. In our study the GGT values alone did not differ between the groups with different insulin sensitivity. Thus, the use of FLI in this context is convenient as the equitation comprises - in addition to GGT - also BMI, waist circumference and triglycerides, parameters not specific to liver function but indicative for insulin resistance of the liver, adipose tissue and skeletal muscle. Furthermore, we noticed less pronounced suppression of plasma FFA release during the OGTT in women with FLI≥60. This agrees with previous studies in NAFLD patients showing not only an impaired suppression of FFA during the OGTT [27] but also after insulin infusion in euglycemic clamp studies [2], [28]. However, hepatic insulin resistance is still of particular interest as a major determinant of impaired fasting glucose indicating a prediabetic state with increased risk for diabetes manifestation. Thus, although an association between NAFLD and insulin resistance is well accepted it still remains unclear whether insulin resistance causes NAFLD or hepatic steatosis per se reduces insulin sensitivity.

As there is growing evidence for NAFLD to actively promote the proinflammatory/prothrombotic state in metabolic syndrome, we additionally assessed the association of inflammatory cytokines and prothrombotic factors with FLI. Our pGDM with FLI≥60 (G3) showed increased values of usCRP and interleukin-6, as well as of PAI-1, t-PA and fibrinogen compared with women in G1 group or NGT. On the other hand, there was no difference in TNF-α concentration between the FLI groups. All these cytokines might be secreted not only by adipose tissue but also by hepatocytes. Thus, elevated levels of these factors could reflect the occurrence of inflammatory processes in NAFLD [4]. Fibrinogen secretion is predominantly influenced by systematic inflammation, whereas PAI-1 is more related to fat accumulation in the liver than in visceral adipose tissues [29], [30]. Therefore, prothrombotic risk profile seems to be partly independent from the contribution of abdominal adiposity. In contrast to Cigolini *et al* [31], we observed higher t-PA levels in patients with risk for fatty liver. This might be explained by the altered risk profile of our study cohort as t-PA was shown to be highly associated with diabetes.

Adiponectin is inversely correlated to hepatic fat content as well as to endogenous glucose production and thus is strongly suggested to represent a link between hepatic fat and IR [32]. Data for leptin levels in patients with NAFLD are more conflicting, but an association with severity of disease is assumed [33]. We observed lower adiponectin and higher leptin levels in the group of FLI values ≥60 and emphasize their potential role in disease pattern of women with GDM.

One of the most important findings of our study is that women with a very recent history of GDM and FLI≥60 had a significantly higher risk for the manifestation of diabetes over 10 years of observation: The association between hepatic steatosis and higher risk for incident diabetes has been recently shown in a large population of subjects without glucose disorder. A Japanese study compared NAFLD patients with normoglycemia to a group with prediabetes and made an analysis over a period of up to 10 years [34], and showed that prediabetes enhances development of type 2 diabetes by about 6.4 times compared to participants with normal glucose level. This association might be of pathogenic importance also in our study cohort as GDM is well recognized for its predisposing character for overt type 2 diabetes. The low affinity of the FLI≤20 group for diabetes manifestation might be explained by their comparable level of insulin sensitivity to the NGT participants of the study. Balkau *et al.* [11] used FLI to calculate the odds ratio for incident type 2 diabetes in a Caucasian population of non-diabetic 1861 men and 1950 women. Men and women with a score of 70 or above had a respective 3.43- and 11.05-fold increased risk for developing type 2 diabetes within 9 years compared to participants with a baseline FLI≤20. Thus, our study further extends previous findings [11], [23] showing for the first time that use of FLI may predict type 2 diabetes in the specific cohort of young women with pGDM.

Moreover, in a recent long-term observational study FLI was validated as a predictor of 15-year all-cause mortality, with independent results to hepatic-related, cardiovascular disease as well as cancer-related mortality rates but with high correlation to IR [35].

The point that we used an index for the diagnosis of fatty liver can be criticised but the gold standard method, which is liver biopsy, is as well not regarded as 100% precise due to sample variability. Performing a biopsy is also very invasive and carries a certain risk, thus only proxy markers are preferably used in epidemiology (i.e. ratio of liver enzymes, ultrasound) [4]. Hepatic fat detection with ultrasound has a threshold of above 30%, while for MR the limit is 5% [8]. We could additionally approve the reliability of FLI by demonstrating a tight but non-linear association to hepatocellular lipid content and suggest that the use of FLI with its specific cut-points is accurate for the estimation of fatty liver prevalence in our cohort. As per definition of the index subjects with higher FLI levels are supposed to have higher BMI levels. Thus this factor might be confounding the results, particularly in relation to parameters of insulin resistance. However, our findings revealed lower oral glucose insulin sensitivity index (OGIS) levels in the group with the highest FLI levels, even after adjustment for BMI and waist.

In summary, we suggest that fatty liver is associated with deterioration of IR as well as with elevated levels of circulating proinflammatory and prothrombotic factors in women with pGDM. Most interestingly, fatty liver was associated with an increased chance to develop manifest diabetes within 10 years of follow-up. Thus, these data strengthen the idea that women with impaired glucose tolerance who accumulate excess fat in the liver are at higher risk for further deterioration of IR and consequently for manifestation of type 2 diabetes as well as cardiovascular disease.
